# Recombinant human C1 esterase inhibitor treatment for hereditary angioedema attacks in children

**DOI:** 10.1111/pai.13065

**Published:** 2019-05-29

**Authors:** Avner Reshef, Vesna Grivcheva‐Panovska, Aharon Kessel, Shmuel Kivity, Maria Klimaszewska‐Rembiasz, Dumitru Moldovan, Henriette Farkas, Vaclava Gutova, Stephen Fritz, Anurag Relan, Bruno Giannetti, Markus Magerl

**Affiliations:** ^1^ Barzilai University Hospital Ashkelon Israel; ^2^ Medical University Skopje Skopje North Macedonia; ^3^ Technion Faculty of Medicine Bnai Zion Medical Center Haifa Israel; ^4^ The Tel Aviv Medical Center Tel Aviv Israel; ^5^ Pediatric Hospital Krakow Poland; ^6^ MediQuest Clinical Research Sangeorgiu de Mures Romania; ^7^ Semmelweis University Budapest Hungary; ^8^ Institute of Immunology and Allergology Pilsen Czech Republic; ^9^ Portland Clinical Research Portland Oregon USA; ^10^ Pharming Healthcare Inc. Bridgewater New Jersey USA; ^11^ Pharming Group NV Leiden The Netherlands; ^12^ Charité Universitätsmedizin Berlin Berlin Germany

**Keywords:** angioedema, child, complement C1 inactivator proteins, complement C1s, hereditary, hereditary angioedema type I and type II, recombinant proteins

## Abstract

**Background:**

Attacks of hereditary angioedema (HAE) due to C1 esterase inhibitor deficiency (C1‐INH‐HAE) usually begin during childhood or adolescence. However, limited data are available regarding indications and modalities of treatment of children. This study evaluated recombinant human C1‐INH (rhC1‐INH) for HAE attacks in children.

**Methods:**

This open‐label, phase 2 study included children aged 2‐13 years with C1‐INH‐HAE. Eligible HAE attacks were treated intravenously with rhC1‐INH 50 IU/kg body weight (maximum, 4200 IU). The primary end‐point was time to beginning of symptom relief (TOSR; ≥20 mm decrease from baseline in visual analog scale [VAS] score, persisting for two consecutive assessments); secondary end‐point was time to minimal symptoms (TTMS; <20 mm VAS score for all anatomic locations).

**Results:**

Twenty children (aged 5‐14 years; 73 HAE attacks) were treated with rhC1‐INH. Seventy (95.9%) of the attacks were treated with a single dose of rhC1‐INH. Seven (35.0%) children were treated for four or more attacks. Overall, median TOSR was 60.0 minutes (95% confidence interval [CI], 60.0‐65.0); data were consistent across attacks. Median TTMS was 122.5 minutes (95% CI, 120.0‐126.0); data were consistent across attacks. No children withdrew from the study due to adverse events. No treatment‐related serious adverse events or hypersensitivity reactions were reported; no neutralizing antibodies were detected.

**Conclusions:**

Recombinant human C1‐INH was efficacious, safe, and well tolerated in children. Data support use of the same dosing regimen for HAE attacks in children (50 IU/kg; up to 4200 IU, followed by an additional dose, if needed) as is currently recommended for adolescents and adults.

**Key Message**

This study demonstrates that weight‐based dosing of recombinant human C1 esterase inhibitor (rhC1‐INH) 50 IU/kg (maximum, 4200 IU) is efficacious, safe, and well tolerated for treating hereditary angio‐oedema (HAE) attacks in children as young as 5 years of age. More than 95% of HAE attacks required only a single dose of rhC1‐INH. This study adds to the overall evidence that rhC1‐INH is efficacious and well tolerated for HAE attacks across various age groups and attack locations.

## 1 INTRODUCTION

Hereditary angioedema (HAE) due to C1 esterase inhibitor (C1‐INH) deficiency (C1‐INH‐HAE) is a rare disorder caused by mutations in the *SERPING*1 gene.^1^ Functional deficiency of the C1‐INH protein leads to disinhibition of the complement^2^ and contact^3^ enzyme cascades, causing overproduction of bradykinin and resulting in increased vascular permeability and fluid leakage to surrounding tissues.^4^, ^5^ HAE is characterized by unpredictable, acute, recurring episodes of angioedema in subcutaneous and/or submucosal tissues. Angioedema episodes can occur in various locations, including the abdomen, periphery, oro‐facial/pharyngeal/laryngeal region, or urogenital region.^1^, ^6^ HAE attacks may be painful and disfiguring, and, in the case of upper airway attacks, potentially life‐threatening.^6^, ^7^ Furthermore, HAE negatively affects patient quality of life and mood (eg, anxiety, depression), both during and between HAE attacks.^8^, ^9^

Onset of HAE attacks typically occurs during childhood or adolescence.^10^ Studies have found mean age of symptom onset to be 4‐14 years,^10^, ^11^, ^12^, ^13^, ^14^, ^15^ with onset by age 10 in approximately 50% of patients.^11^, ^16^, ^17^ Early symptom onset is often associated with a more severe disease course.^11^, ^13^, ^16^ This is particularly troubling because diagnosis of HAE has been reported to be a median of 8.5 years from time of symptom onset.^18^

International consensus recommendations delineate acute treatment of HAE attacks in a pediatric population.^10^, ^19^ In principle, shorter time to treatment of attacks has been shown to improve clinical outcomes.^19^, ^20^ Despite progress made during the last decade with introduction of novel treatments for HAE, there is a paucity of evidence‐based treatment options for children.^10^ Recombinant human C1 esterase inhibitor (rhC1‐INH) is among the options for adults and adolescents. It is purified from the milk of rabbits^5^ and approved in multiple countries for treatment of HAE attacks. The efficacy and safety of rhC1‐INH for management of HAE attacks have been demonstrated in adolescents and adults in randomized, placebo‐controlled trials,^21^, ^22^ open‐label extension studies,^23^, ^24^, ^25^ and pooled analyses.^26^ In addition, data published in 2017 showed rhC1‐INH to be efficacious and well tolerated as prophylactic therapy in individuals aged 13 years or older with C1‐INH‐HAE.^27^ The objective of the current study was to evaluate the efficacy and safety of rhC1‐INH for acute treatment of HAE attacks in children.

## 2 METHODS

### 2.1 Study design

This open‐label, phase 2, multicenter, multinational clinical study was conducted from January 2012 to July 2017 at 18 centers in 10 countries (ClinicalTrials.gov identifier: NCT01359969). This study was conducted in accordance with the International Conference on Harmonisation Good Clinical Practice guidelines, the ethical principles of the Declaration of Helsinki, and applicable local regulatory requirements. Study protocols were approved by an institutional review board or independent ethics committee at each site. All patients provided assent to study participation, and a parent/legal guardian provided written informed consent before study procedures were initiated.

### 2.2 Study participants

Children aged 2‐13 years with a clinical and confirmed laboratory diagnosis (C1‐INH activity <50% of normal) of C1‐INH‐HAE were eligible for the study. Key exclusion criteria included a diagnosis of acquired C1‐INH deficiency, allergy to rabbits, and medical history of 10 HAE attacks previously treated with study medication. An attack was eligible for treatment if the patient presented to the site with an onset of attack symptoms within 5 hours of evaluation, had at least one anatomic location involved, and had an investigator rating of at least moderate intensity (≥3; range, 0‐5) at presentation, with no indication of spontaneous regression.

### 2.3 Intervention

Eligible patients received within 5 minutes an intravenous injection of rhC1‐INH (Ruconest^®^; Pharming Technologies B.V.) 50 IU/kg of body weight (maximum dose, 4200 IU). One additional dose could be administered at the investigator's discretion, based on clinical response. No more than two doses were to be administered within 24 hours. Additional medications for treatment of HAE (eg, analgesics, antiemetics, fluid replacement) were permitted post‐treatment, if needed, at the investigator's discretion. In case of lack of treatment success, rescue therapy (eg, plasma‐derived C1 esterase inhibitor [pdC1‐INH] or fresh frozen plasma) could be administered according to local clinical standards. Patients remained at the study site for at least 4 hours post‐treatment. After discharge, scheduled follow‐ups included a post‐treatment telephone contact at 24 hours ± 4 hours and clinic visits on Days 28 and 90.

### 2.4 Assessments

The 100‐mm visual analog scale (VAS) was completed by the patient, or parent/legal guardian if needed, at baseline and 0.5, 1, 2, 4, 8, and 24 hours post‐treatment. The baseline VAS assessment was to be completed immediately prior to treatment with study medication. Patients (or parent/legal guardian) were required to fill out the VAS forms for severity of angioedema symptoms in five anatomic locations: abdominal, urogenital, oro‐pharyngeal/laryngeal, facial, and peripheral. Investigators rated HAE attack severity using a 6‐point scale (0 = no symptoms to 5 = life‐threatening) at baseline and 0.5, 1, 2, and 4 hours post‐treatment (investigator score [IS]). Functional C1‐INH concentration was assessed using blood samples collected prior to administration of study medication, at 5 minutes post‐treatment, and 2‐4 hours post‐treatment for the first HAE attack. Functional C1‐INH concentrations were expressed as percentage of normal (set as 100%), based on a pool of plasma collected from healthy humans, and C1‐INH concentrations below the lower limit of quantification (24%) were estimated as 12%.

The primary efficacy end‐point was time to beginning of symptom relief (TOSR), defined as the interval during which the VAS score decreased by 20 mm or more from baseline, with this decrease persisting for two consecutive VAS assessments. The secondary efficacy end‐point was time to minimal symptoms (TTMS), defined as the interval during which the VAS score had decreased to less than 20 mm (ie, fell below 20 mm on the 100‐mm VAS) for all anatomic locations of the attack (ie, clinical remission). Exploratory efficacy end‐points included TOSR using the IS (defined as the first time‐point at which the IS decreased from baseline by one point or more at any eligible location), TTMS using the IS (defined as score ≤1 at all HAE attack locations assessed), and time to complete resolution of symptoms at all locations based on diary results (ie, the patient recorded when [date and time] all angioedema symptoms at all locations had resolved)*.*

Treatment‐emergent adverse events (AEs), defined as events that occurred or increased in intensity from first rhC1‐INH administration up to 97 days post‐treatment, were recorded. Safety assessments also included physical examination, vital signs, electrocardiogram, laboratory parameters, and immunogenicity testing. AEs of special interest included serum antibodies against rhC1‐INH (immunoglobulin [Ig]M and IgG classes) and against impurities arising from rabbit milk (ie, host‐related impurities) using enzyme‐linked immunosorbent assay tests for plasma samples obtained at baseline and Days 28 and 90 post‐treatment for each attack.

### 2.5 Statistics

No formal sample size was calculated a priori, and study enrollment continued until at least 20 patients were treated for at least one HAE attack. The intent‐to‐treat (ITT) population included all patients who received at least one dose of study medication; the efficacy population included all patients in the ITT population who had efficacy data; and the pharmacokinetic/pharmacodynamic population included all patients in the ITT population with sufficient laboratory data. Unless otherwise specified, data were analyzed using descriptive statistics. Kaplan‐Meier analyses were performed for each of the time‐to‐event end‐points, and patients who did not achieve the outcome of interest were censored at the time of their last available assessment. Data were analyzed using SAS^®^ versions 9.3 and 9.4 (SAS Institute Inc.).

## 3 RESULTS

### 3.1 Patient population

Of 57 children screened and considered eligible for treatment, 20 received rhC1‐INH for at least one HAE attack and were included in the ITT and efficacy populations (Table 1). Patient ages were 5‐14 years, with a similar percentage of males and females. The 20 children who received rhC1‐INH were treated for 73 HAE attacks; seven (35.0%) children were treated for four or more HAE attacks. The most common locations for eligible attacks were abdominal (n = 39, 53.4%), peripheral (n = 15, 20.6%), facial (n = 9, 12.3%), urogenital (n = 7, 9.6%), and oro‐pharyngeal/laryngeal (n = 5, 6.9%), with two attacks involving multiple locations (one attack abdominal and peripheral; and one attack oro‐pharyngeal/laryngeal and facial). Fifteen children (75.0%) completed the study. Five children discontinued study participation due to consent withdrawal: one patient left the country, one patient was no longer interested in continuing, one patient's mother decided not to attend additional study visits for personal reasons, one patient preferred to treat HAE attacks at home with a different medication, and one patient was no longer considered a child based on age and weight (ie, obese).

**Table 1:** Baseline demographics at presentation of attack 1

Parameter	Patients (n = 20)
Age, y, mean (SD)	8.2 (2.9)
Range	5.0‐14.0^a^
Male, n (%)	11 (55.0)
Race, n (%)	
White	19 (95.0)
Black	1 (5.0)
Weight, kg, mean (SD)	34.8 (20.6)
Range	16.0‐93.1
History of HAE attacks/y	
Mean (SD)	21.3 (21.1)
Range	0‐80
Current prophylactic therapy, n (%)	0 (0)
Lifetime occurrence of oro‐pharyngeal/laryngeal HAE attacks, mean (range)	
Mild or moderate^b^	0.7 (0‐6)
Severe	0.5 (0‐3)

Abbreviations: HAE, hereditary angioedema; SD, standard deviation.

a Children were eligible to enter the study before reaching the age of 13 y but could have presented with the first HAE attack past the age of 13 y.

b n = 19.

### 3.2 Efficacy

**Figure 1:**
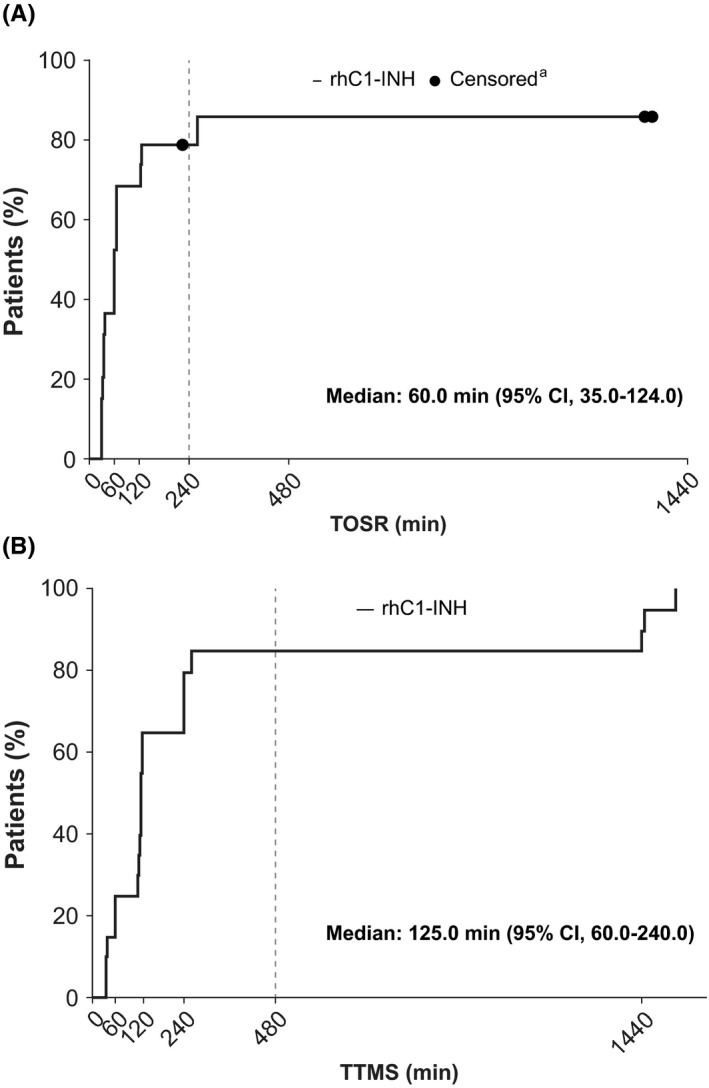
Kaplan‐Meier plots of TOSR (A) and TTMS (B) based on VAS scores for the first HAE attack. CI, confidence interval; HAE, hereditary angioedema; rhC1‐INH, recombinant human C1 esterase inhibitor; TOSR, time to beginning of symptom relief; TTMS, time to minimal symptoms; VAS, visual analog scale. ^a^Patients who did not meet end‐point during follow‐up were censored at last available VAS assessment time‐point

Seventy (95.9%) of 73 HAE attacks were treated with a single dose. One child received a second dose for HAE attacks 3 and 8, and one child received a second dose for attack 3. Median TOSR based on VAS score (primary end‐point) for the 73 HAE attacks was 60.0 minutes (95% confidence interval [CI], 60.0‐65.0 minutes). For the first HAE attack, the median TOSR was 60.0 minutes, and most children had TOSR within 4 hours (240 minutes; Figure 1A). Data were consistent across individual HAE attacks, with a median TOSR of approximately 60.0 minutes post‐treatment and overlapping 95% CIs (Table 2). Median TTMS based on VAS score (secondary end‐point) for the 73 HAE attacks was 122.5 minutes (95% CI, 120.0‐126.0 min). For the first HAE attack, median TTMS was 125.0 minutes, and most of the 20 children had minimal symptoms (ie, clinical remission) within 8 hours (480 minutes; Figure 1B). Across individual HAE attacks, median TTMS was consistent, at approximately 120 minutes post‐treatment with overlapping 95% CIs (Table 2).

**Table 2:** Time to beginning of symptom relief (TOSR)^a^ and time to minimal symptoms (TTMS)^b^ based on VAS, by HAE attack

HAE attack	TOSR (min)		TTMS (min)	
	Patient, n	Median (95% CI)	Patient, n	Median (95% CI)
All attacks	20	60.0 (60.0‐65.0)	20	122.5 (120.0‐126.0)
Attack 1	19^c^	60.0 (35.0‐124.0)	20	125.0 (60.0‐240.0)
Attack 2	11^c^	60.0 (30.0‐120.0)	12	122.0 (60.0‐245.0)
Attack 3	9	62.0 (30.0‐75.0)	9	120.0 (35.0‐485.0)
Attack 4	6^c^	61.5 (30.0‐125.0)	7	120.0 (30.0‐125.0)
Attack 5	6	60.0 (30.0‐65.0)	6	120.0 (60.0‐495.0)
Attack 6	5	65.0 (31.0‐240.0)	5	126.0 (120.0‐240.0)
Attack 7	4	63.5 (60.0‐120.0)	4	180.0 (120.0‐487.0)
Attack 8	4	60.0 (60.0‐66.0)	4	124.5 (60.0‐241.0)
Attack 9	3	60.0 (36.0‐60.0)	3	120.0 (60.0‐249.0)
Attack 10	3	120.0 (30.0‐128.0)	3	120.0 (60.0‐485.0)

Abbreviations: CI, confidence interval; HAE, hereditary angioedema; VAS, visual analog scale.

a Defined as interval during which VAS score decreased by ≥20 mm from baseline at any eligible anatomic location, with this decrease persisting for two consecutive assessments.

b Defined as interval during which VAS score decreased to <20 mm for all anatomic locations in which VAS scores were recorded.

c For attacks 1, 2, and 4, a patient received treatment prior to VAS assessment completion. Thus, there was no baseline VAS score to determine efficacy for these attacks.

Consistent with the results of the VAS‐based analyses, median TOSR based on IS for the 73 HAE attacks was 60.0 minutes (95% CI, 40.0‐60.0 minutes), and median TTMS based on IS was 126.0 minutes (95% CI, 120.0‐240.0 minutes). Median time to complete resolution of symptoms (based on patient scores) at all locations for the 73 HAE attacks was 262.5 minutes (95% CI, 220.0‐535.0 minutes). Three of the 73 HAE attacks (4.1%; n = 2 children) received a second dose of rhC1‐INH. Two of these occurred during the first HAE attack, for which TOSR using VAS score occurred more than 4 hours post‐treatment. One of these same children experienced acute tonsillitis with concomitant pharyngitis during his second HAE attack (abdominal) and received paracetamol (a disallowed medication) within 45 minutes of rhC1‐INH administration. This child was subsequently treated successfully for attacks 3 and 4 during the study.

### 3.3 Functional C1‐INH concentrations

Functional C1‐INH concentrations (percentage of normal) were measured during the first HAE attack. Nineteen children had baseline functional C1‐INH concentrations below the lower limit of quantification (<24.0% of normal), and one child had a baseline value of 35% of normal. For the 19 children who received a single dose of rhC1‐INH and had a blood sample collected at 5 minutes post‐treatment, normal functional C1‐INH concentrations were observed at 5 minutes post‐treatment (median, 122%; range, 62%‐168% of normal). Nineteen children still had higher‐than‐baseline functional C1‐INH data 2‐4 hours post‐treatment (median, 41%; range, 12%‐81%), while one child had a functional C1‐INH concentration below the lower limit of quantification. Overall, 18 of 19 children with data for both post‐treatment assessments had concentrations of functional C1‐INH greater than 70% of normal (lower limit of normal range) at 5 minutes and/or 2‐4 hours post‐treatment.

### 3.4 Safety

Adverse events were reported in 11 (55.0%) of the 20 children, with the most common AEs being naso‐pharyngitis (n = 3; 15.0%), vomiting (n = 3; 15.0%), abnormal blood lymphocyte morphology (n = 2; 10.0%), and viral infection (n = 2; 10.0%). The two instances of abnormal blood lymphocyte morphology were considered by investigators to be possibly related to study medication. The events were considered mild in intensity and resolved without intervention (ie, self‐limited). One child experienced two such events at 38 and 55 days, respectively, after dosing with rhC1‐INH for HAE attack 4; this child received treatment for eight HAE attacks, with no indication of recurrence of abnormal lymphocyte morphology with additional treatments. One child experienced one event 31 days after dosing with rhC1‐INH for HAE attack 1, and one event 10 days after dosing with rhC1‐INH for HAE attack 2; this child received treatment for six HAE attacks, with no indication of recurrence of abnormal lymphocyte morphology with additional treatments. Based on these events being self‐limited, resolving spontaneously, and not recurring upon re‐treatment, the authors speculate that these events were probably related to a viral infection.

There was no evidence of an increase in incidence of AEs in children treated for more than one HAE attack. In addition, most AEs were mild in intensity, with no evidence of a relationship between HAE attack number and AE intensity (Table 3). Two children had a severe AE (abdominal pain and vomiting, respectively); both AEs were considered unrelated to study medication. No hypersensitivity reactions or drug‐related serious AEs were reported. Sporadic, transient immune responses to rhC1‐INH and host‐related impurities were observed but were not temporally associated with clinical AEs. No evidence of neutralizing antibodies to C1‐INH was observed in any child treated with rhC1‐INH at any time during the study. There were no clinically significant changes in vital signs or electrocardiogram changes during the study. No deaths or discontinuations due to AEs were reported during the study.

**Table 3:** Incidence of AEs^a^ by maximum intensity

HAE attack	Patients with AE intensity, n (%)			
	Mild	Moderate	Severe	Any
Attack 1 (n = 20)	6 (30.0)	0	2 (10.0)^b^	8 (40.0)
Attack 2 (n = 12)	1 (8.3)	1 (8.3)	0	2 (16.7)
Attack 3 (n = 9)	1 (11.1)	1 (11.1)	0	2 (22.2)
Attack 4 (n = 7)	2 (28.6)	1 (14.3)	0	3 (42.9)
Attack 5 (n = 6)	0	0	0	0
Attack 6 (n = 5)	0	0	0	0
Attack 7 (n = 4)	0	1 (25.0)	0	1 (25.0)
Attack 8 (n = 4)	1 (25.0)	0	0	1 (25.0)
Attack 9 (n = 3)	0	0	0	0
Attack 10 (n = 3)	0	1 (33.3)	0	1 (33.3)

Abbreviations: AE, treatment‐emergent adverse event; HAE, hereditary angioedema.

a AEs reported >97 d after drug administered were not considered treatment‐emergent.

b One AE of abdominal pain in one patient and one AE of vomiting in one patient; both AEs were considered unrelated to study drug.

## 4 DISCUSSION

Although onset of HAE symptoms typically occurs during late childhood and early adolescence, there are limited data published on efficacy and safety of modern therapies for children with HAE. The current study supports the safety and efficacy of rhC1‐INH for acute treatment of HAE attacks in children as young as 5 years of age. Treatment with rhC1‐INH 50 IU/kg provided rapid TOSR (eg, median time of 1 hour) and was consistently efficacious across multiple HAE attacks. Most of the evaluable children with rhC1‐INH and post‐treatment pharmacokinetic data through 2‐4 hours had concentrations of functional C1‐INH greater than 70% of normal. Furthermore, based on pharmacokinetic modeling with subcutaneous pdC1‐INH,^28^ the median functional C1‐INH level of greater than 41% observed in the current study (3‐4 times baseline levels) at 2‐4 hours post‐treatment would be considered effective. Administration of rhC1‐INH was safe and well tolerated for treatment of multiple HAE attacks, and no thromboembolic, hypersensitivity, or anaphylactic AEs were observed. Limitations of the study included the open‐label, non‐randomized design, lack of control group (eg, placebo), and small sample size. Previous, larger, randomized, placebo‐controlled studies have demonstrated the efficacy and safety of rhC1‐INH in patients aged 12 years and older,^21^, ^22^ and the current study showed that rhC1‐INH is well tolerated and efficacious for treatment of HAE attacks in pediatric patients aged 5 to 14 years. Thus, the findings of this open‐label study are in line with previous clinical studies of rhC1‐INH.

In several countries, formulations of pdC1‐INH are indicated for HAE attacks in children aged 12 years and younger.^4^ A systematic review of pdC1‐INH for HAE attacks in pediatrics, including children less than 12 years of age, reported efficacy and safety results similar to those observed in adults.^29^ Ecallantide, a plasma kallikrein inhibitor indicated for acute treatment in patients who are at least 12 years of age, exhibited a safety and efficacy profile^30^ similar to that observed in adults with HAE. Results of an open‐label, phase 3 study of icatibant reported efficacy and tolerability of this bradykinin B2 receptor antagonist for acute treatment of C1‐INH‐HAE in pediatric patients (inclusion criteria, aged 2‐17 years).^31^ However, icatibant is only indicated as acute treatment in adults.

Recombinant human C1‐INH was developed to be a safe and effective alternative to pdC1‐INH for acute treatment of HAE attacks. Advantages of rhC1‐INH include consistent supply (not dependent on availability of human donor plasma) and lack of risks related to human bloodborne pathogens. Multiple trials have shown that treatment with rhC1‐INH significantly and rapidly improves HAE attack symptoms among adolescents and adults with C1‐INH‐HAE.^21^, ^22^, ^23^, ^24^, ^25^, ^26^ Results of the current study are consistent with the rhC1‐INH treatment response in adolescents and adults and support the use of the same dosing regimen for HAE attacks in children (50 IU/kg; maximum, 4200 IU) as currently recommended for adolescents and adults, followed by an additional dose, if needed.

In conclusion, in this study of a pediatric population, rhC1‐INH 50 IU/kg (maximum, 4200 IU) was efficacious, safe, and well tolerated in treating HAE attacks in children with C1‐INH‐HAE. More than 95% of HAE attacks required only a single dose of rhC1‐INH and, overall, findings in children align with published data for adults, indicating that rhC1‐INH for HAE attacks is efficacious and well tolerated across various age groups and attack locations.

## CONFLICT OF INTERESTS

Avner Reshef reports receiving honoraria from CSL Behring and receiving research grants to institution funding from BioCryst Pharmaceuticals, Inc., CSL Behring, Pharming Group NV, Shire HGT, Stallergenes Greer, and Teva. Vesna Grivcheva‐Panovska reports serving as principal investigator for clinical trials sponsored by Pharming Group NV. Aharon Kessel reports serving as principal investigator for clinical trials sponsored by Pharming Group NV. Shmuel Kivity reports serving as principal investigator for clinical trials sponsored by Pharming Group NV. Maria Klimaszewska‐Rembiasz reports serving as principal investigator for clinical trials sponsored by Pharming Group NV. Dumitru Moldovan reported having ties to BioCryst Pharmaceuticals, Inc., CSL Behring, Pharming Technologies BV, Shire HGT, and Swedish Orphan Biovitrum AB. Henriette Farkas reports serving as principal investigator for clinical trials sponsored by Pharming Group NV, and receiving consultancy/speaker fees and honoraria from BioCryst Pharmaceuticals, Inc., CSL Behring, Pharming Group NV, and Shire HGT. Vaclava Gutova reports serving as principal investigator for clinical trials sponsored by Pharming Group NV. Stephen Fritz reports serving as principal investigator for clinical trials sponsored by Pharming Group NV. Anurag Relan reports being an employee of Pharming Healthcare, Inc. Bruno Giannetti reports being an employee of Pharming Group NV. Markus Magerl reports receiving honoraria from BioCryst Pharmaceuticals, Inc., CSL Behring, Pharming Group NV, and Shire HGT, and serving as principal investigator for clinical trials sponsored by BioCryst Pharmaceuticals, Inc., CSL Behring, Pharming Group NV, and Shire HGT.

## AUTHOR CONTRIBUTIONS

A Reshef, VG‐P, A Relan, and BG were involved in the conception and design of the study. A Reshef, VG‐P, AK, SK, MK‐R, DM, HF, VG, SF, and MM were investigators in the study and involved in data acquisition and interpretation. All authors critically reviewed, revised, and approved the manuscript for publication and agreed to be accountable for all aspects of the work in ensuring that questions related to the accuracy or integrity of any part of the work are appropriately investigated and resolved.
